# Successful medical treatment of a hepatic pregnancy: a case report

**DOI:** 10.1186/s13256-017-1227-1

**Published:** 2017-03-15

**Authors:** A. Sibetcheu Tchatou, R. Tchounzou, L. Mbuagbaw, E. T. Mboudou

**Affiliations:** 1 Faculty of Medicine and Biomedical Sciences, University of Yaounde I, Yaounde, Cameroon; 2 Gynaecology and Obstetrics Unit, Douala Gynaeco-Obstetric and Paediatric Hospital, Douala, Cameroon; 3 Department of Clinical Epidemiology and Biostatistics, McMaster University, Hamilton, Canada

**Keywords:** Hepatic pregnancy, Methotrexate, Case report, Cameroon

## Abstract

**Background:**

Hepatic pregnancy is a rare form of abdominal pregnancy, often documented only as case reports.

**Case presentation:**

We report here the case of a 24-year-old African woman, gravida 4 para 3, presenting with right upper quadrant pains and metrorrhagia after amenorrhea of 8 weeks 5 days. Elements in favor of the diagnosis of hepatic pregnancy were her clinical presentation, the kinetics of β-human chorionic gonadotropin titers, and the presence of a sub-hepatic mass on ultrasound. We successfully treated this patient with intramuscular methotrexate only.

**Conclusions:**

The interest of this case resides in the rarity of this condition and the therapeutic approach used. Clinicians should raise their index of suspicion for hepatic pregnancy when faced with females of reproductive age with such a clinical presentation.

## Background

Abdominal pregnancies constitute 1.4 % of ectopic pregnancies. This rare form of ectopic pregnancy is associated with high mortality due to diagnostic challenges and the massive intraperitoneal hemorrhage caused by the removal of the placenta [1]. A hepatic location is extremely rare and described only as case reports. In these circumstances, treatment options are not as well defined as they are with tubal ectopic pregnancy. We present this case of hepatic pregnancy diagnosed with non-invasive explorations and medical treatment.

## Case presentation

A 24-year-old African woman, gravida 4 para 3, presented with a 2-week history of right upper quadrant pain and an 8 weeks and 5 days amenorrhea with spotting. She first consulted in a secondary health facility where her clinical evaluation revealed a hepatomegaly on abdominopelvic ultrasound and positive urinary pregnancy test without intrauterine or tubal gestational sac. These findings motivated the medical team to refer her to our tertiary center.

This was her first episode of such pain. She had no history of sexually transmitted diseases and no history of contraception. She had never had an operation. Her hepatitis B and C status were unknown. There was no dizziness, asthenia, or jaundice.

Her vital signs were stable: blood pressure of 120/60 mm Hg, pulse rate of 70 beats per minute, respiratory rate of 16 cycles per minute, and temperature at 36.5 °C. She weighed 59 kg. She was not pale. Her right upper quadrant was tender on deep palpation but not after deep breath. There was no guarding or rebound tenderness. Her uterus was of normal size and there were no adnexal masses noted. Her rectovaginal pouch was free and non-tender. We suspected acute cholecystitis or acute viral hepatitis in pregnancy.

On another abdominopelvic ultrasound, a heterogeneous poorly vascularized mass was visible under the right lobe of her liver, of size 42 × 38 mm. There was no hemoperitoneum and her uterus was empty without adnexal masses. On full blood count, there was mild microcytic and hypochromic anemia at 10.2 g per dL, with normal leukocyte and platelet counts. Her liver transaminases and renal function were normal. Her serum β-human chorionic gonadotropin (βhCG) titers rose from 200 mUI/mL to 3000 mUI/mL in 48 hours. No gestational sac was visible on concomitant pelvic ultrasound.

Our final diagnosis was hepatic pregnancy. We chose to administer 1 mg/kg of methotrexate intramuscularly since she was hemodynamically stable and the Fernandez score [2] was in favor of medical treatment. Evolution was favorable and she was discharged at day 5. Two weeks later, her serum βhCG titer was 15 mUI/mL. She did not show up at 1-month appointment.

## Discussion

Abdominal pregnancy is a rare form of ectopic pregnancy. It occurs in 9.2 per 1000 ectopic pregnancies [1]. Location in the liver is extremely rare. Using “hepatic pregnancy”, “hepatic ectopic pregnancy”, and “primary hepatic pregnancy” as keywords in PubMed, only 21 cases have been reported in the English literature of which four were in Africa [3–6], and of the 21 reported cases, one had medical treatment only [7].

Of these 21 cases, 61.9 % of cases presented with abdominal pain and signs of hemoperitoneum on admission or during hospitalization. Other nonspecific signs included localized abdominal tenderness (right upper quadrant, right flank), spotting, and right upper quadrant masses with amenorrhea. These misleading signs may point clinicians toward digestive and hepatobiliary pathologies, thus delaying the diagnosis.

Abdominal ultrasound is a first-line medical imaging modality in the diagnosis of ectopic pregnancy. However, the accuracy is better with magnetic resonance imaging (MRI), especially in describing the regional anatomy, the precise location of the placenta, and key elements to determine whether the placenta should be removed or not [8]. Ultrasounds coupled with the βhCG titers were the only available and affordable means of diagnosis used for this patient. The site mostly described in the literature is under the right lobe of the liver, as in our case [5–7].

The management of hepatic pregnancies is variable. Surgery, either laparotomy or laparoscopy, is the most described and used therapeutic option. The main challenge with this technique is the cataclysmic hemorrhage that can occur during surgery. One patient died of multiorgan failure secondary to an uncontrollable bleeding from the placenta site left *in situ* [9]. Common surgical procedures used include hepatic artery ligation, lobectomy, omental transplantation, and liver packing. Other authors administered a direct injection of methotrexate during a diagnostic laparotomy [10] or a combination of preoperative arterial embolization, laparoscopic extraction of the fetus, and postoperative administration of methotrexate for the placenta left *in situ* [11].

The hemodynamic stability of our patient and the favorable Fernandez score prompted us to choose the least invasive approach, administration of methotrexate intramuscularly at a dose of 1 mg/kg. This dose was repeated 7 days later, as a precaution since a 15 % reduction in βhCG titers had been obtained. This medical approach is similar to what Shippey *et al*. did [7].

## Conclusions

As clinicians, we should always rule out hepatic pregnancy when faced with such a clinical presentation. Intramuscular injection of methotrexate to the mother is the least invasive treatment option reported in the treatment of hepatic pregnancies. It could be a safe therapeutic option in uncomplicated forms of this rare condition.
